# Saliency-based 3D convolutional neural network for categorising common focal liver lesions on multisequence MRI

**DOI:** 10.1186/s13244-021-01117-z

**Published:** 2021-11-24

**Authors:** Shu-Hui Wang, Xin-Jun Han, Jing Du, Zhen-Chang Wang, Chunwang Yuan, Yinan Chen, Yajing Zhu, Xin Dou, Xiao-Wei Xu, Hui Xu, Zheng-Han Yang

**Affiliations:** 1 Department of Radiology, Beijing Friendship Hospital, Capital Medical University, No. 95 Yongan Road, Xicheng District, Beijing, 100050 People’s Republic of China; 2 Department of Radiology, Weihai Municipal Hospital, Cheeloo College of Medicine, Shandong University, Weihai, Shandong Province People’s Republic of China; 3 Center of Interventional Oncology and Liver Diseases, Beijing Youan Hospital, Capital Medical University, Beijing, People’s Republic of China; 4 SenseTime Research, SenseTime, Shanghai, People’s Republic of China; 5 WCH-SenseTime Joint Lab, SenseTime, Shanghai, Sichuan People’s Republic of China; 6 SenseBrain Technology, SenseTime, Princeton, NJ 08540 USA

**Keywords:** Deep learning, MRI, Classification, Focal liver lesion, Model interpretation

## Abstract

**Background:**

The imaging features of focal liver lesions (FLLs) are diverse and complex. Diagnosing FLLs with imaging alone remains challenging. We developed and validated an interpretable deep learning model for the classification of seven categories of FLLs on multisequence MRI and compared the differential diagnosis between the proposed model and radiologists.

**Methods:**

In all, 557 lesions examined by multisequence MRI were utilised in this retrospective study and divided into training–validation (*n* = 444) and test (*n* = 113) datasets. The area under the receiver operating characteristic curve (AUC) was calculated to evaluate the performance of the model. The accuracy and confusion matrix of the model and individual radiologists were compared. Saliency maps were generated to highlight the activation region based on the model perspective.

**Results:**

The AUC of the two- and seven-way classifications of the model were 0.969 (95% CI 0.944–0.994) and from 0.919 (95% CI 0.857–0.980) to 0.999 (95% CI 0.996–1.000), respectively. The model accuracy (79.6%) of the seven-way classification was higher than that of the radiology residents (66.4%, *p* = 0.035) and general radiologists (73.5%, *p* = 0.346) but lower than that of the academic radiologists (85.4%, *p* = 0.291). Confusion matrices showed the sources of diagnostic errors for the model and individual radiologists for each disease. Saliency maps detected the activation regions associated with each predicted class.

**Conclusion:**

This interpretable deep learning model showed high diagnostic performance in the differentiation of FLLs on multisequence MRI. The analysis principle contributing to the predictions can be explained via saliency maps.

**Supplementary Information:**

The online version contains supplementary material available at 10.1186/s13244-021-01117-z.

## Key points

- AI has the potential to relieve physicians by automating the process.
- This model could accurately classify common liver masses on multisequence MRI.
- Different MRI scanners and liver background did not affect the model performance.
- Saliency maps could explain model decision-making and let radiologists verify the diagnosis.

## Introduction

With the development of imaging technology, most focal liver lesions (FLLs) can be detected accurately by MRI [1]. Nevertheless, diagnosing FLLs with imaging alone remains a challenge. The imaging features of FLLs are diverse and complex, and different lesion features overlap. Atypical characteristics in some common lesions make the diagnosis challenging, including atypical morphologic features, atypical location or lesions that may mimic other primary liver tumours [2]. Maximising the imaging diagnosis accuracy of FLLs is paramount in avoiding unnecessary biopsies [3] and optimal patient management. Meanwhile, the evaluation and analysis of medical images are generally subjective and may possibly be affected by the experience of radiologists with various levels of specialisation [4]. Artificial intelligence (AI) could aid doctors in diagnosing FLLs and potentially be useful in both helping inexperienced physicians and bridging the gap between novice and expert radiologists [5].

As a strategy for AI, convolutional neural network (CNN)-based deep learning systems have been widely considered in radiology [6, 7]. Different from radiologists who diagnose diseases through radiological features and conventional machine learning algorithms that rely on handcrafted features, CNNs can automatically learn complex features from medical images [8]. Therefore, by learning from a sufficiently large amount of training data, CNNs may accurately categorise FLLs without relying on the experience of radiologists.

There have been several AI studies on FLL classification, but they have mainly focused on two-dimensional (2D) CNN models [9, 10] or have been based on computed tomography (CT) images [11]. There is a lack of research on three-dimensional (3D) CNNs based on MRI data. Compared with 2D CNNs, 3D CNNs based on magnetic resonance imaging (MRI) data can resample tumour slices more effectively, improve the sample size, obtain rich 3D tumour information and capture tissue characteristics more accurately [12, 13]. In addition, previous studies still lack interpretability for the “black box”. At present, the output of a 3D CNN heat map is still technically difficult to interpret, which makes it impossible to verify whether the model follows some aspects of human expert knowledge. The aim of our study was to potentially relieve physicians and staff of the need to carry out primary diagnosis by automating the process and thus lighten the burden on radiologists. We developed an interpretable 3D CNN based on multiple sequences for the classification of seven categories of common FLLs and validated its performance compared to radiologists with various levels of specialisation.

## Material and methods

This retrospective study was approved by the institutional Ethical Review Committee (Approval Number: 2019-P2-230-01) and the requirement for written informed consent was waived. In this study, a sample size was not prespecified. Nonemployee or nonconsultant authors analysed and controlled the data.

### Patients and diseases

There were 445 study patients, each with one anonymised liver MRI (study) acquired between January 2017 and December 2019. Studies were obtained from our institution’s picture archiving and communication system (PACS) according to the procedures detailed in Additional file 1: Fig. S1.

The inclusion criteria were as follows: (1) participants underwent unenhanced and enhanced liver MRI inspection; (2) participants had one of the following common FLLs, including liver cyst, cavernous haemangioma (HEM), hepatic abscess (HEP), focal nodular hyperplasia (FNH), hepatocellular carcinoma (HCC), intrahepatic cholangiocarcinoma (ICC) and hepatic metastasis (MET); and (3) up to one imaging study per patient was included, and up to six lesions were used in each study. We chose only one type of lesion from each case for the following evaluations. The exclusion criteria were as follows: (1) patients with MRI studies of insufficient image quality. (2) Participants who had received treatment related to the lesion before MRI inspection; and (3) diffuse lesions for which the boundary could not be delineated or malignancies involving the portal vein, hepatic vein or adjacent organs. Most malignant tumours were confirmed by histopathology, while other malignancies and benign tumours were diagnosed by follow-up reports that were supported by two radiologists (with 10 and 20 years of experience, respectively) for 3–12 months.

### MRI acquisition protocol

Abdominal MRI was performed on the patient in a supine position using 1.5-T and 3-T MRI scanners, including Siemens (Prisma, TrioTim), GE Healthcare (GE 750w, Signa) and Philips (Ingenia) systems. T2-weighted imaging (T2WI), diffusion-weighted imaging (DWI) (*b* value: 800 s/mm^2^) and apparent diffusion coefficient (ADC) mapping from standard institutional liver MRI protocols were performed with acquisition times of 2–2.5 min. All the unenhanced sequences and enhanced phases were acquired according to the institutional liver MRI protocol. Imaging parameters varied across different scanners and time frames. The parameters used to acquire the images are summarised in Table 1. Contrast-enhanced T1WI sequences were used with acquisition times of 12–18 s. All patients underwent MRI using gadobenate dimeglumine, which was intravenously injected at a dosage of 0.1 mmol/kg (maximum dose, 20 mL) and a rate of 2 mL/s followed by a normal saline flush (50 mL at 2 mL/s). Except for precontrast T1WI, T2WI, DWI and ADC mapping, postcontrast images were analysed, including the late arterial phase (LAP, 15–20 s postinjection), portal venous phase (PVP, 60–70 s postinjection) and delayed phase (DP, 3–5 min postinjection).

**Table 1:** Image acquisition

MRI scanners	Sequences		
3.0 T MRI			
	TR, T2WI	DWI *b* = 0.800	LAVA/ VIBE/ eTHRIVE
TR (ms)	2–3respratory cycles	3000	Minimum
TE (ms)	85	Minimum	Minimum
Flip angle (°)	150	90	15
Matrix	288 × 224	128 × 128	288 × 172–320 × 216
FOV (mm)	380–420	380–420	380–420
Echo train length	16	128	–
Thickness, mm	6–8	6–8	3–4

*TR* respiratory triggered, *TE* echo time

### Model construction and evaluation based on CNN algorithm

There were two groups of classification tasks. The first group of tasks was to classify all the tumours into two categories: benign and malignant. The second group of tasks divided the lesions into seven categories as follows: 0, cyst; 1, FNH; 2, haemangioma; 3, abscess; 4, HCC; 5, ICC; and 6, metastasis. Here, 0, 1, 2 and 3 indicate benign lesions, while 4, 5 and 6 indicate malignancies. A multitask framework has been demonstrated to improve learning efficiency, potential prediction accuracy and overfitting problems for task-specific models.

#### Data pre-processing

MR images were downloaded from the PACS and stored as Digital Imaging and Communications in Medicine (DICOM) format. The images were then converted to NIFTI format to hide patient information. The liver tumours were manually segmented by two subspecialty-trained radiologists with an average of 9-year experience in abdominal diagnosis. The outline of tumour on all sequences was drawn in our self-developed module in the 3D-Slicer software (version 4.8.1, Harvard University, Boston, MA, USA). It could provide more valuable information of the tumour region. When there was a discrepancy between the two radiologists on whether the region was a lesion or on the lesion type, a joint review was performed until a consensus was reached for a final decision.

#### Image processing

Seven-sequence (T2WI, DWI, ADC, T1WI, LAP, PVP, DP) images and the matched annotated images were resampled at the same voxel spacing of [1, 1, 1] with the nearest neighbour interpolation algorithm. We normalised the intensity of MRI images to the range of [0, 1]. For lesions with different diameters, the cropping strategy was different. Lesions smaller than 16 mm were directly cropped to 32 * 32 * 32 mm, lesions larger than 16 mm but smaller than 32 mm were cropped to 64 * 64 * 64 mm, and the remaining lesions were cropped by dilating the area of the lesion. (The dilation size was randomly changed during the training phase.) Then, the cropped patch images with the target lesion and the matched annotated images were resised to 64 * 64 * 64 (mm) using bilinear interpolation and the nearest neighbour interpolation algorithms. The cropped annotated images were processed using the Gaussian blur method. Online data augmentation was applied, which included rotation, flipping, shifting, scaling, lighting alteration and Gaussian noise addition.

#### Model training

**Fig. 1:**
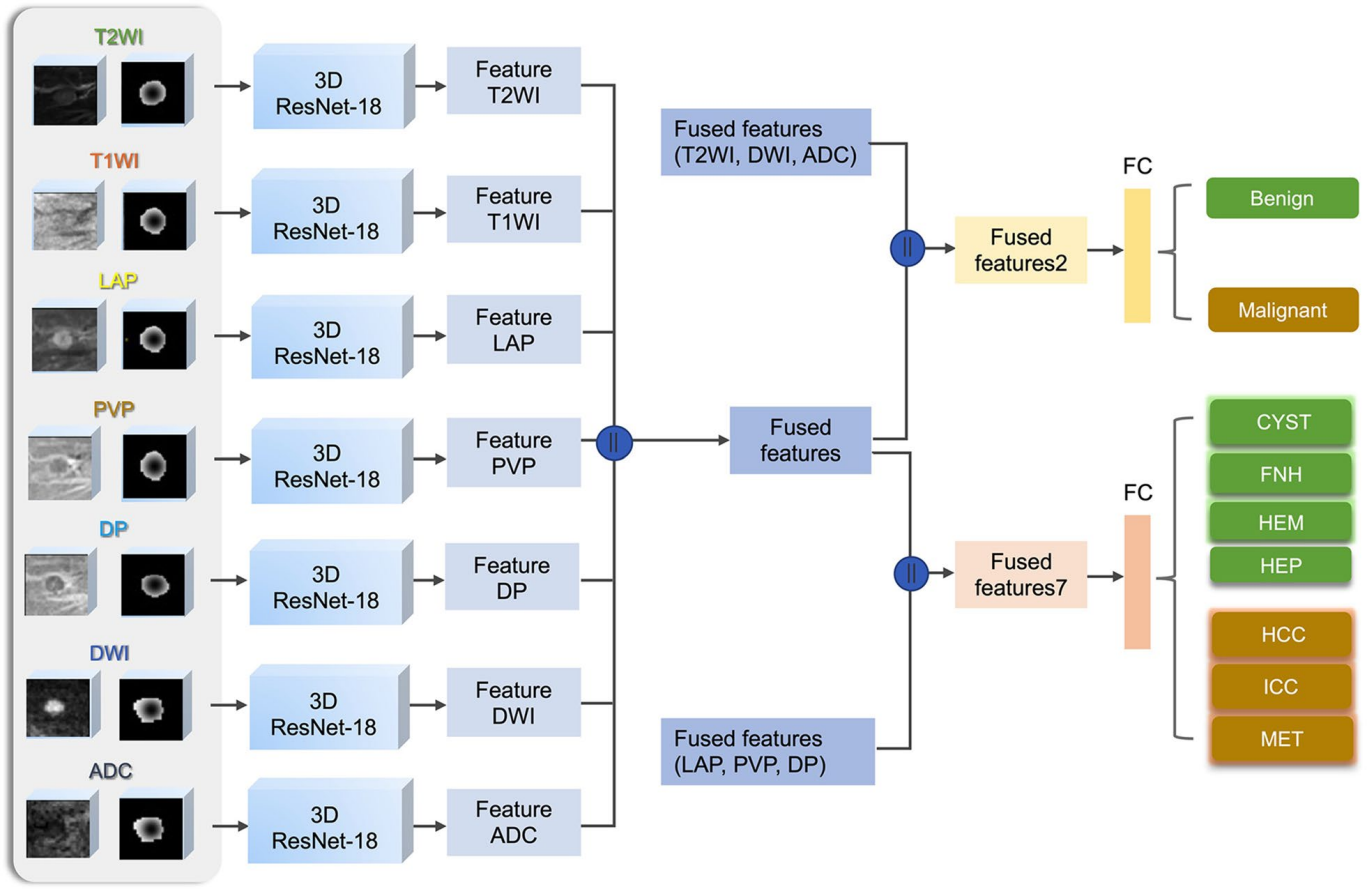
Architecture of the proposed deep learning model

Our deep learning model was trained on a GeForce GTX 1080Ti (NVIDIA) graphic processing unit using Python 3.7 and PyTorch 1.4.0. The architecture of the model is illustrated in Fig. 1. We applied a 3D extension of the 2D ResNet-18 as the backbone [14]. For each sequence, the MRI image and the matched Gaussian annotated image were fed into a 3D ResNet-18 network, and then a feature representation was obtained. All seven feature representations were concatenated as one fused feature. The feature representations of T2WI, DWI and ADC images were concatenated as one fused feature, which, together with the fused feature of all seven sequences, was used for binary classification (fused feature 2). The feature representations of LAP, PVP and DP images were concatenated as another fused feature, which, together with the fused feature of all seven sequences, was used for the seven-way classification (fused feature 7). To obtain the classification results of the two tasks, both fused feature 2 and fused feature 7 were input into fully connected (FC) layers. The cross-validation method split the dataset of the development cohort into training and validation datasets, and fivefold cross-validation was used.

#### Model evaluation

The performance of the model was evaluated on an unseen test dataset containing 113 lesions. For each of the five experiments, the model showing the best performance on the validation dataset was selected. The five selected models were used to infer the two-way (benign and malignant) and seven-way classification of the test data. The average predicted result of the five experiments on the test data served as the final result. The general demographics of the patients, lesion type, lesion size and MRI scanner were also analysed. We evaluated the influence of liver background (cirrhosis and fatty liver) on the model classification performance. Finally, the model outputs saliency maps to explain the analysis principle.

### Comparison to radiologist performance

Test data were anonymised and independently presented to three categories of radiologists, including two radiology residents (with 1 and 2 years of experience), two general radiologists (with 8 and 12 years of experience) and two academic radiologists (with 17 years and 22 years of experience). All doctors routinely read liver MRI images. ITK-SNAP (version 3.6.0, www.itksnap.org) was used to read images that contained lesions and their surrounding liver parenchyma. Radiologists were given the same MRI sequences available to the AI system.

### Statistical analysis

The characteristics of the development and test datasets are described as follows: continuous variables are expressed as the mean ± SD or as the median with interquartile range (IQR) according to the normality of the data; categorical variables are expressed as frequencies (percentage, %). Student’s *t* test or Kruskal–Wallis test was used for continuous variables, and Chi-square test or Fisher exact test was used for categorical variables.

The area under the receiver operating characteristic (ROC) curve (AUC), accuracy, sensitivity, specificity, positive predictive value (PPV), negative predictive value (NPV), positive diagnostic likelihood ratio (PLR) and negative diagnostic likelihood ratio (NLR) were also calculated. Additionally, 95% confidence intervals (CIs) were calculated with the modified Wilson method. Different liver backgrounds were compared using Pearson’s chi-squared test and Fisher’s exact test. The accuracy with 95% CI was used to compare the difference in diagnostic performance between the model and the radiologists. Interreader agreement was assessed using Cohen’s kappa statistic. Confusion matrices were plotted to evaluate the strengths and weaknesses of the model and the radiologists. *p* < 0.05 was regarded as statistically significant.

## Results

### Patient demographics

A total of 445 participants were divided into development (*n* = 356) and test (*n* = 89) datasets. There was no significant difference in age (*p* = 0.795) or sex (*p* = 0.647) between the development and test datasets, nor was there a significant difference in tumour type, size or MRI scanner (*p* > 0.05) (Table 2).

**Table 2:** Data and patient characteristics

Characteristic	Total	Development dataset	Test dataset	*p* value
No. of patients	445	356	89	
Age (year)	58	58	57	0.795
Median (IQR)	(48.00, 64.00)	(47.00, 64.00)	(51.00, 63.00)	
*Sex*				0.647
Male	272 (100%)	221 (84.4%)	50 (15.6%)	
Age (year)	58	58	59	0.238
Median (IQR)	(49.25, 64.00)	(47.75, 64.00)	(54.00, 65.75)	
Female	183 (100%)	144 (78.7%)	39 (21.3%)	
Age (year)	56	57	56	0.329
Median (IQR)	(46.00, 63.00)	(47.00, 63.00)	(37.50, 62.50)	
Lesion number (%)	557	444 (79.7%)	113 (20.3%)	
Lesion diameter	26.8	27.3	24	0.418
Median (IQR)	(16.80, 41.50)	(16.75, 41.70)	(17.10, 36.38)	
*Lesion type*				0.907
Cyst				
Lesion number (%)	86 (100%)	70 (80.7%)	16 (19.3%)	
Lesion diameter	12.8	12.75	12.8	0.898
Median (IQR)	(8.65, 22.50)	(8.30, 23.43)	(9.80, 15.50)	
*Haemangioma*				
Lesion number (%)	101 (100%)	79 (78.2%)	22 (21.8%)	
Lesion diameter	19.7	19.5	20.1	0.815
Median (IQR)	(15.30, 32.60)	(15.55, 32.65)	(15.05, 25.02)	
*FNH*				
Lesion number (%)	57 (100%)	46 (80.7%)	11 (19.3%)	
Lesion diameter	34.13 ± 12.89	35.35 ± 13.24	28.79 ± 10.28	0.148
Mean ± SE				
*Abscess*				
Lesion number (%)	88 (100%)	67 (76.1%)	21 (23.9%)	
Lesion diameter	62.98 ± 31.26	65.20 ± 32.23	56.31 ± 29.10	0.479
Mean ± SE				
*HCC*				
Lesion number (%)	78 (100%)	63 (80.7%)	15 (19.3%)	
Lesion diameter	28.9	29.35	26.8	0.537
Median (IQR)	(23.20, 45.90)	(23.45, 46.65)	(22.00, 38.20)	
*ICC*				
Lesion number (%)	70 (100%)	59(84.3%)	11 (15.7%)	
Lesion diameter	52.45	51.15	63.55	0.323
Median (IQR)	(30.72, 69.45)	(29.43, 66.65)	(47.12, 94.50)	
*Metastasis*				
Lesion number (%)	77 (100%)	60 (77.9%)	17 (22.1%)	
Lesion diameter	28.55	28.8	28.1	1
Median (IQR)	(20.18, 38.32)	(19.55, 38.75)	(27.80, 28.80)	
*Manufacturer and model*				0.499
GE Signa	126 (100%)	99 (78.6%)	27 (21.4%)	
GE Discovery MR750w	62 (100%)	46 (69.7%)	16 (30.3%)	
Philips Ingenia	58 (100%)	50 (86.2%)	8 (13.8%)	
Siemens prisma	50 (100%)	42 (84.0%)	8 (16%)	
Siemens TrioTim	149 (100%)	119 (79.9%)	30 (20.1%)	

*IQR* interquartile range

### Deep learning model performance

**Fig. 2:**
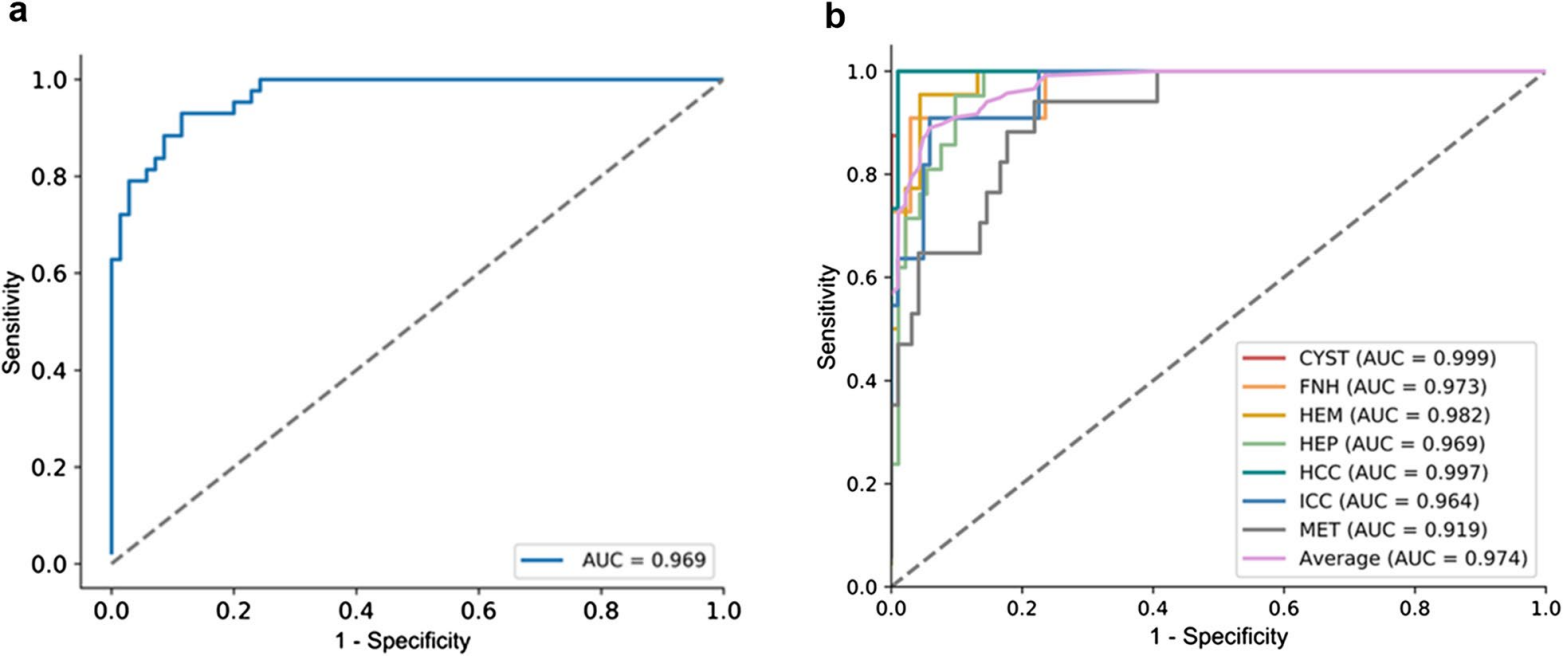
ROC curves of the (**a**) two-way and (**b**) seven-way deep learning model classification

The model showed high performance in the test dataset with 113 lesions, with an average AUC of 0.969 in the two-way classification and 0.919 (0.857–0.980) to 0.999 (0.996–1.000) in the seven-way classification (Fig. 2). The accuracy, sensitivity, specificity, PPV, NPV, PLR and NLR for each lesion category, determined using the test data, are described in Table 3. The model was found to perform well in diagnosing cysts and HCCs, with median accuracies of 0.991 (0.951, 1.000) and 0.991 (0.952, 1.000), but poorly in diagnosing metastases and abscesses, with median accuracies of 0.805 (0.723, 0.868) and 0.885 (0.813, 0.932). The median model sensitivity for the seven categories ranged from 0.909 (0.623–0.995) to 1.000 (0.806–1.000), the specificity ranged from 0.781 (0.689–0.852) to 0.990 (0.944–0.999), and the PPV ranged from 0.432 (0.287–0.591) to 0.941 (0.730–0.997).

**Table 3:** Deep learning model diagnostic performance in the two-way and seven-way classification on the test dataset

	Two-way classification	Seven-way classification						
		Cyst	FNH	Haemangioma	Abscess	HCC	ICC	Metastasis
ACC (95% CI)	0.903 (0.834, 0.945)	0.991 (0.951, 1.000)	0.965 (0.913, 0.986)	0.956 (0.901, 0.981)	0.885 (0.813, 0.932)	0.991 (0.952, 1.000)	0.938 (0.878, 0.970)	0.805 (0.723, 0.868)
Sensitivity (95% CI)	0.930 (0.814, 0.976)	1.000 (0.806, 1.000)	0.909 (0.623, 0.995)	0.955 (0.782, 0.998)	1.000 (0.845, 1.000)	1.000 (0.796, 1.000)	0.909 (0.623, 0.995)	0.941 (0.730, 0.997)
Specificity (95% CI)	0.886 (0.790, 0.941)	0.990 (0.944, 0.999)	0.971 (0.917, 0.990)	0.956 (0.892, 0.983)	0.859 (0.773, 0.916)	0.990 (0.944, 0.999)	0.941 (0.878, 0.973)	0.781 (0.689, 0.852)
PPV (95% CI)	0.833 (0.704, 0.913)	0.941 (0.730, 0.997)	0.769 (0.497, 0.918)	0.840 (0.653, 0.936)	0.618 (0.450, 0.761)	0.938 (0.717, 0.997)	0.625 (0.386, 0.815)	0.432 (0.287, 0.591)
NPV (95% CI)	0.954 (0.873, 0.984)	1.000 (0.962, 1.000)	0.990 (0.946, 0.999)	0.989 (0.938, 0.999)	1.000 (0.954, 1.000)	1.000 (0.962, 1.000)	0.990 (0.944, 0.999)	0.987 (0.929, 0.999)
PLR (95% CI)	8.140 (4.218, 15.706)	97.000 (13.802, 681.695)	30.909 (9.981, 95.722)	21.716 (8.294, 56.860)	7.077 (4.076, 11.712)	98.000 (13.943, 688.794)	15.455 (60,955, 340,342)	4.303 (2.895, 6.395)
NLR 95% CI)	0.079 (0.026, 0.235)	0.000 (0.000, –)	0.094 (0.014, 0.607)	0.048 (0.007, 0.323)	0.000 (0.000, –)	0.000 (0.000, –)	0.097 (0.015, 0.626)	0.075 (0.011, 0.506)

*ACC* accuracy, *PLR* positive likelihood ratio, *NLR* negative likelihood ratio, *NPV* negative predictive value, *PPV* positive predictive value

There were no effects of liver background on model performance. In the two-way classification of the model, the accuracy rates in patients with and without liver cirrhosis in the test cohort were 100.0% and 87.0%, respectively (*p* = 0.358). The accuracy rates in patients with and without fatty liver were 95.7% and 86.7%, respectively (*p* = 0.401). In the seven-way classification of the model, the accuracy rates in patients with and without liver cirrhosis in the test cohort were 100.0% and 77.0%, respectively (*p* = 0.116). The accuracy rates in patients with and without fatty liver were 65.2% and 83.3%, respectively (*p* = 0.102).

### Deep learning model performance compared to radiologist performance

**Fig. 3:**
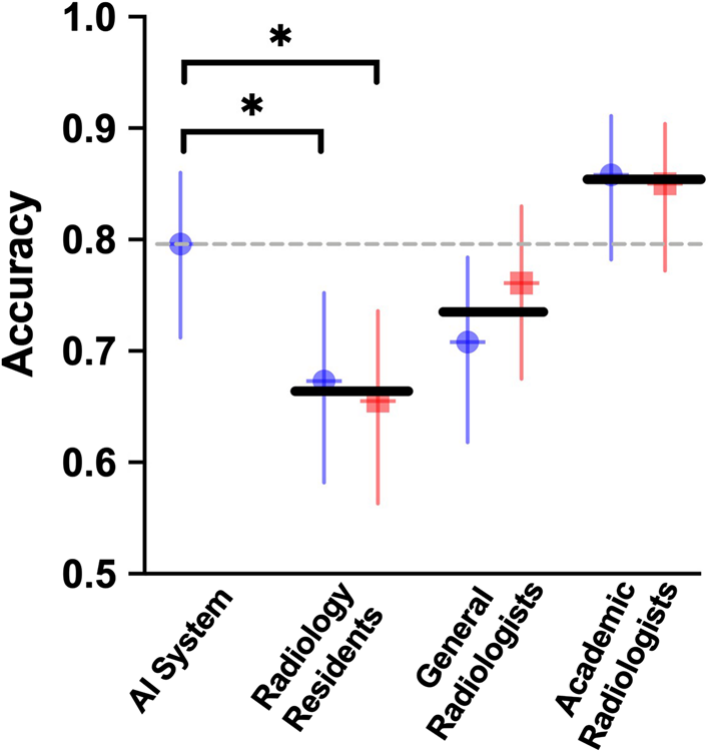
Performance of the model compared to that of radiologists with various levels of specialisation. Performance is measured as accuracy. Each point represents a single radiologist, and the horizontal line represents the mean across each radiologist category. The horizontal dashed line is the performance of the model. Error bars represent 95% binomial probability CIs. *There was a significant difference in performance between the model and two types of residents (*p* < 0.05)

We compared the performance of the model with that of three categories of radiologists. The performance of the model (90 lesions correct of 113 lesions; mean correct percentage across participants, 79.6%) was better than that of the radiology residents (76–78 lesions correct of 113 lesions [67–69%; mean correct percentage across participants, 68%]) (*p* = 0.035) and general radiologists (80–88 lesions correct of 113 lesions [71–78%; mean correct percentage across participants, 74%]) (*p* = 0.346). The accuracy of the model was lower than that of the academic radiologists (96–98 lesions correct of 113 lesions [85–87%; mean percent correct across participants, 86%]) (*p* = 0.291) (Fig. 3). There was a statistically significant difference in diagnostic performance between the model and radiology residents but not between the model and general or academic radiologists. The agreement was then measured by comparing radiologists within the same specialisation level (Table 4). Two radiologists in the same category showed good consistency (kappa > 0.75, *p* < 0.01).

**Table 4:** Consistency evaluation between radiologists in the same category

	Kappa	*Z* score	*p* value
Radiology residents	0.915	22.49	< 0.01
General radiologists	0.776	18.867	< 0.01
Academic radiologists	0.802	20.471	< 0.01

### Evaluating radiologist and model errors

**Fig. 4:**
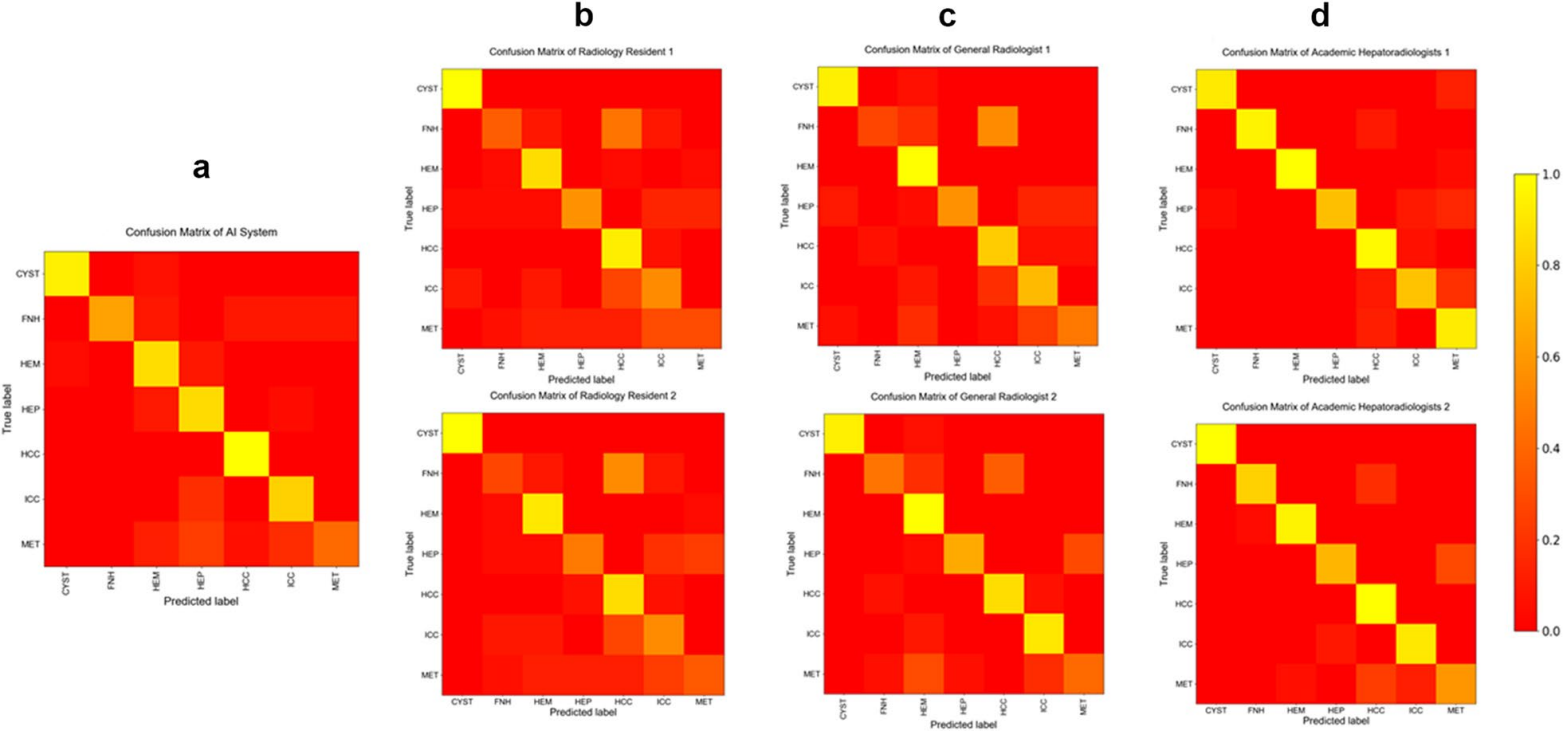
Confusion matrices showing the distribution of the diagnostic predictions of seven categories of common FLLs for the (**a**) model, (**b**) radiology residents, (**c**) general radiologists and (**d**) academic radiologists. True disease labels are shown along the *x*-axis, and the predictions are shown along the *y*-axis. Predictions the same as the true disease labels resulted in yellow squares along the diagonal from top left to bottom right. Each category of radiologists made errors in divergent diagnoses, but fewer errors occurred with increasing radiologist specialisation

We evaluated the differential diagnosis distribution of the model and radiologists by means of confusion matrices. Confusion matrices between the model and academic radiologists resembled each other, but in some cases, the model and radiologists made different types of errors. Fewer errors occurred with increasing radiologist specialisation. Radiologists with various levels of specialisation may mistake one certain type of lesion for another. All radiologists made errors in some cases of HCC, but the model did not make the same error (Fig. 4). The average number of model errors was 23. The model performed well in diagnosing HCC without any mistakes. The model performed poorly in diagnosing FNH among benign lesions and metastasis among malignant lesions. Of 17 metastasis cases, 10 were misidentified, 4 of which were identified as abscesses.

### Saliency map

**Fig. 5:**
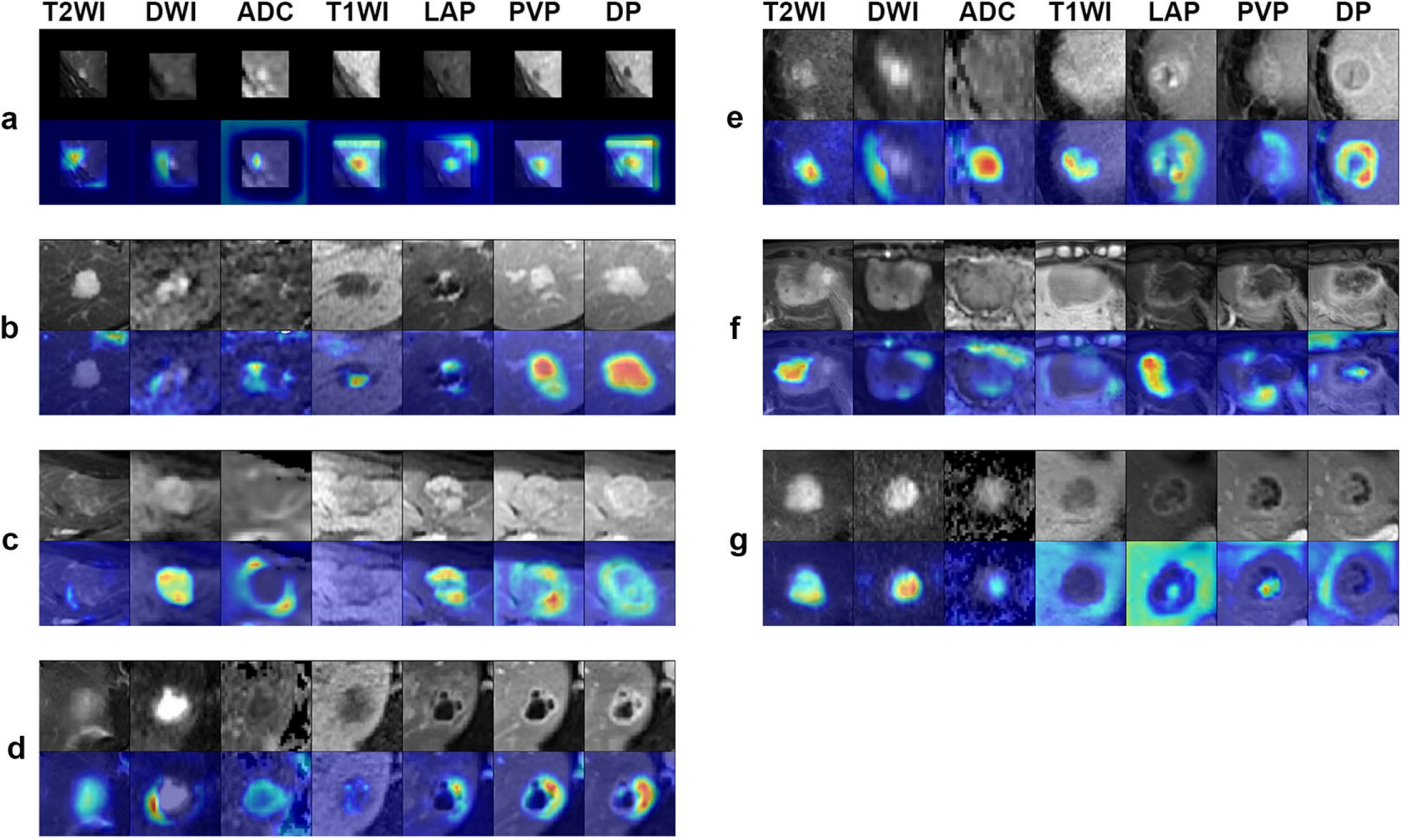
Saliency map for example images from 7 category classifications of the test set. Red highlights the activation region of the radiologic imaging feature more associated with the predicted class. (**a**) Cyst, (**b**) HEM, (**c**) FNH, (**d**) HEP, (**e**) HCC, (**f**) ICC and (**g**) MET gain good display, and these maps reveal some typical radiologic imaging features of different FLLs

We selected example saliency maps from seven categories of the test set. Red highlights the activation region of the radiologic imaging feature more associated with the predicted class (Fig. 5). For cysts, the model focused on T2 hyperintensity and T1 hypointensity without contrast enhancement. For haemangioma, the model fixes its attention on discontinuous peripheral nodular enhancement, which progresses in a centripetal direction. For FNH, the model focused on intense arterial hyperenhancement, with near isointensity on the PV and slow gradual enhancement of the central scar. For abscesses, the model focused on restricted diffusion and the typical pattern of peripheral enhancement. For HCC, the model directed its attention to strong arterial enhancement, washout on PVP and DP and capsular enhancement on DP. For ICC, the model directed its attention to rim arterial phase hyperenhancement and delayed central enhancement. For MET, the model concentrated on restricted diffusion and rim hyperenhancement.

**Fig. 6:**
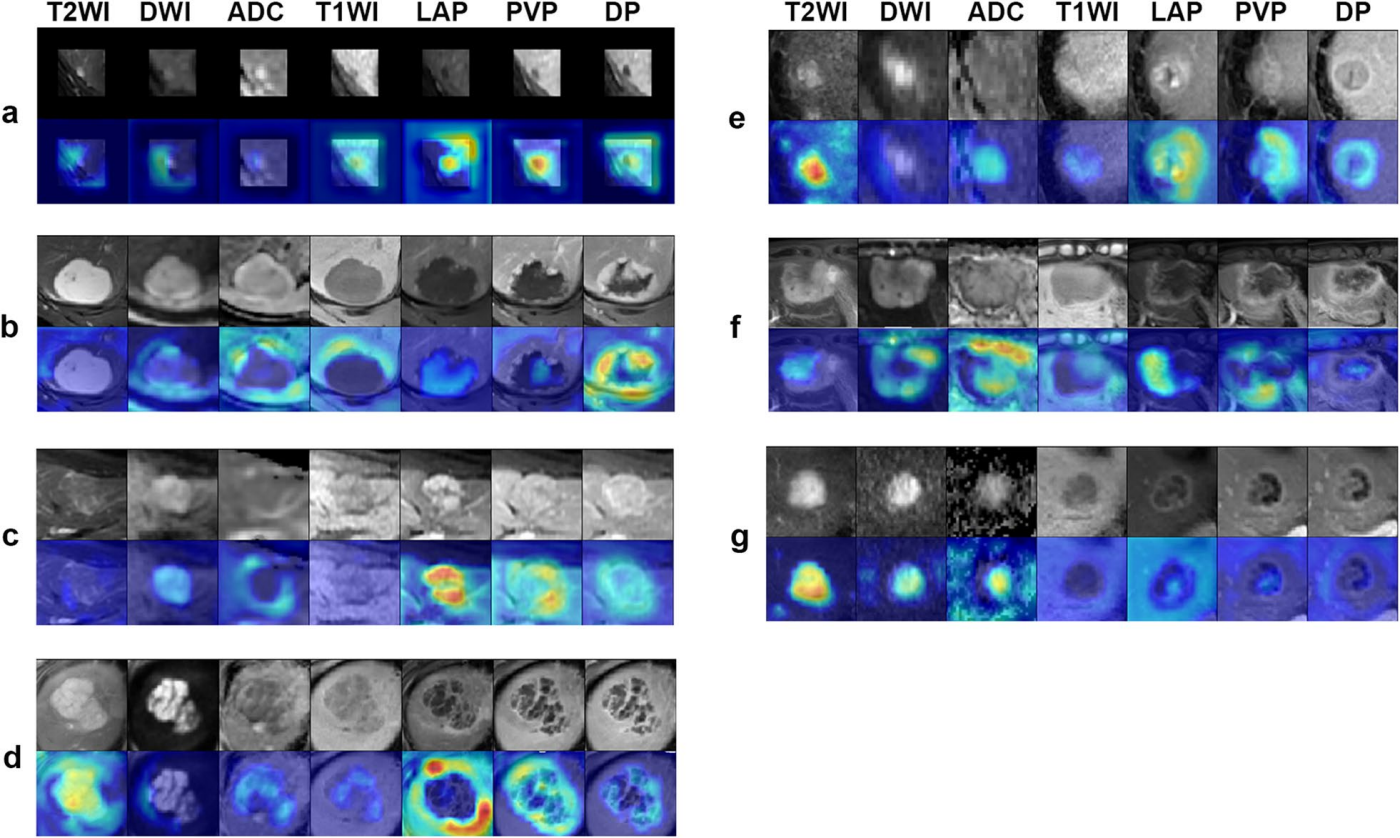
Saliency map for example images from 7 category classifications of the test set. Red highlights the activation region of the weight of each sequence/phase in differential diagnosis. (**a**) Cyst, (**b**) HEM, (**c**) FNH, (**d**) HEP, (**e**) HCC, (**f**) ICC and (**g**) MET show good display, and these maps reveal that the importance of different sequences/phases in the analysis of each category of FLLs is different

Figure 6 shows the weight of each sequence/phase in differential diagnosis. Red colour in saliency maps highlights more important sequence/phase. The importance of each sequence/phase in the analysis of seven categories of FLLs is variable.

## Discussion

We developed an interpretable 3D CNN-based classification model for seven categories of common FLLs, using seven sequences and outputted saliency maps to interpret the principle of the model decision-making. The model showed good performance, with an AUC of 0.969 in two-way classification and from 0.919 (0.857–0.980) to 0.999 (0.996–1.000) in seven-way classification.

The accuracy of our model was higher than that of the radiologist residents but slightly lower than that of the academic radiologists. The evaluation of medical images by radiologists is subjective and possibly influenced by personal experience. To improve the accuracy and consistency of radiologists and reduce the variability of interpretation, the Liver Imaging Reporting and Data System (LI-RADS) is constantly updated [15–20]. Even so, the diagnostic consistency among radiologists is still variable [21–24]. Kierans et al. [24] demonstrated that by using LI-RADS 2017, the interreader agreement for major features was moderate (*k* = 0.661), and ancillary features were poor to fair (*k* = 0.257–0.436) [24]. Our model might maintain diagnostic consistency, help inexperienced physicians, improve the diagnostic accuracy of general radiologists and radiology residents, reduce the workload of academic radiologists and bridge the diagnostic gap between novice and expert radiologists and among different hospitals.

Our model showed good performance in diagnosing HCC, while radiologists with various levels of specialisation made a certain number of errors. Radiologists misclassified HCC lesions with unclear washout as FNH or ICC lesions and HCC lesions with faint enhancement as metastases. The model could correctly identify HCC lesions by learning from the images. Saliency maps showed arterial enhancement on LAP, wash out on PVP and DP, and an enhancing capsule on DP, which was consistent with the major imaging features of LI-RADS. This result indicates that AI could aid radiologists and reduce the occurrence of misdiagnosis in clinical work. Meanwhile, saliency maps could help radiologists verify the predictions of the model and help clinicians understand the model performance.

The model showed poor performance in abscesses and metastases. A saliency map of abscesses showed that the model fixed its attention to the pattern of peripheral enhancement. However, there were many overlapping imaging features between abscesses and malignancies (Additional file 1: Fig. S2). In addition, with the progression of abscesses, there will be a variety of imaging features [25]. Primary malignancies with different biological behaviours and pathological changes along with tumour growth will affect the imaging features of metastases. Because the metastases in our study had different origins, the features were different [26]. The enhancement pattern of metastases is affected by nodule size, tumour vascularity and pathological behaviour changes with tumour growth. Small metastases may show hyperenhancement, while larger tumours may show intranodular necrosis or vascular thrombosis [27]. Hence, the model showed poor performance in small abscesses and metastases. In addition, a few typical lesions were misclassified by the model, which indicates that the performance of the model still has room for improvement.

Confusion matrices showed that the sources of diagnostic errors for the model and the academic radiologists for each disease were similar. To maintain consistency with the model, the radiologists diagnosed the lesions by reading the images, including the lesions and the surrounding part of the liver parenchyma, without reference to the related medical history or laboratory test results, which might affect the diagnostic accuracy. Radiologists could improve the accuracy by referring to clinical information. Therefore, we speculate that if clinical information and laboratory test results were included in the training process of the model, the diagnosis accuracy and the reliability of interpretation could also be effectively improved.

Our model is based on 3D-CNN. 2D-CNN is based on the assumption that the lesion grows and shrinks in a symmetrical and spherical manner, which is not accurate [28]. 3D CNN can accurately reflect the actual size of the tumour [29], evaluate the asymmetry of the tumour morphology [30] and learn the tissue characteristics of the lesion on MRI. In addition, our model was trained with seven-sequence images that were obtained from 5 types of MRI scanners and included more than 500 lesions. Fatty liver and cirrhosis did not affect the model performance. The model was reliable, robust and predictive. Although the total number of lesions was lower than that in a previous study [9], the model still showed similar performance. In addition, our network involved an attention mechanism, in which the boundary of the tumour was given more attention and then the network learned more representative features to achieve better diagnostic performance. A multitask framework was applied in our study to improve the learning efficiency, potential prediction accuracy and overfitting problems.

The previous study did not include the image information of unenhanced sequences such as T2WI, DWI and ADC. A comprehensive liver MRI protocol needs to evaluate the parenchyma, vasculature and biliary system, which is accomplished by way of a combination of unenhanced sequences and enhanced phases [31]. T2WI with fat suppression represents information about fluid content and fibrotic tissue and increases lesion conspicuity [32]. DWI and ADC can detect and characterise focal liver lesions and evaluate posttreatment changes in the tumour microenvironment [33]. T1WIs are acquired, which provide information regarding the T1 characteristics of lesions and serve as a baseline to evaluate enhanced phases [34]. The lack of unenhanced imaging could not fully evaluate the characteristics of FLL.

Although the classification models in the previous study had good performance, they were difficult to visualise and interpret. However, it is critical to explain model decision-making and let radiologists and clinicians verify the diagnosis [35]. Hence, we straightforwardly displayed interesting slices of 3D feature maps on the image containing the maximum area of the lesion and generated a radiologic imaging feature-based saliency map and sequence/phase weight-based saliency map. The radiologic imaging feature-based saliency map highlights the activation region of the radiologic imaging feature more associated with the predicted class. The sequence/phase weight-based saliency map highlights which image feature is more advantageous in classification by evaluating the importance of each sequence/phase. We showed that applying visualisation methods is important to understand the decisions of the model and is a step that is crucial to increase clinical impact and trust in deep learning models.

Our study has several limitations. First, our study focused on seven common categories of FLLs, while the types of FLLs in clinical practice are more extensive. Deep learning requires a large number of samples for model training. Due to the small number of some type of FLLs, it is difficult to train the model. Therefore, we only included 7 types of common FLLs for a preliminary model to reduce the daily work burden of radiologists. In the future, more patients with different types of FLLs (such as cirrhosis nodules and other rare liver tumours) need to be included to render the model applicable for different disease spectra in clinical practice. We will add validation datasets obtained from external centres to make the model more generalisable and reliable. Second, metastases from different primary origins in our study had different imaging features. Therefore, the model could not learn the lesion characteristics well, and its performance was poor. In the future, we need to increase the number of metastases in training or categorise them by their sources. Third, our study was a single-centre study, and we only used one type of intravenous contrast agent, which may limit the applicability of the model. In the future, we need to collect images from different hospitals using different contrast agents to make the model widely applicable. Fourth, saliency maps only evaluated the importance of a single sequence/phase in diagnosis but did not evaluate sequence/phase combinations. In the process of diagnosis, we need to consider the characteristics of lesions reflected by different sequences and phases on MRI. Therefore, we will continue to analyse the importance of sequence/phase combinations in the future.

## Conclusion

This interpretable deep learning model showed high diagnostic performance in the differentiation of liver masses on multisequence MRI and used a saliency map to explain the analysis principle contributing to predictions, which made it more reliable. Due to the increasing demand for medical imaging in clinics and the different levels of radiologists in different regions, we expect that deep learning models could reduce the daily workload and may be in demand in radiology departments [36].

## Supplementary Information

**Additional file 1: Figure S1**. Flowchart showing study selection according to the inclusion and exclusion criteria, from initial patient search to training-validation and test dataset randomisation. **Figure S2** Axial MRI of a 55-year-old man with an abscess. (**a**) T2WI showed an irregularly shaped, hyperintense neoplasm in segment V. The lesion showed (**b**) hyperintensity on DWI (**c**) with a low ADC, (**d**) hypointensity on T1WI, and (**e**) targetoid rim enhancement in the LAP, (**f**) PVP and (**g**) DP. The lesion was mistaken as ICC by the AI model.

## Data Availability

The datasets used or analysed during the current study are available from the corresponding author on reasonable request.

## Abbreviations

3D: Three-dimensional
ADC: Apparent diffusion coefficient
AI: Artificial intelligence
AUC: Area under the receiver operating characteristic curve
CIs: Confidence intervals
CNN: Convolutional neural network
CT: Computed tomography
DP: Delayed phase
DWI: Diffusion-weighted imaging
FC: Fully connected layers
FLL: Focal liver lesions
FNH: Focal nodular hyperplasia
HCC: Hepatocellular carcinoma
HEM: Cavernous haemangioma
HEP: Hepatic abscess
ICC: Intrahepatic cholangiocarcinoma
IQR: Interquartile range
LAP: Late arterial phase
LI-RADS: Liver imaging reporting and data system
MET: Hepatic metastasis
MRI: Magnetic resonance imaging
NLR: Negative diagnostic likelihood ratio
NPV: Negative predictive value
AP*ACS: Picture archiving and communication system
PLR: Positive diagnostic likelihood ratio
PPV: Positive predictive value
PVP: Portal venous phase
ROC: Receiver operating characteristic
T2WI: T2-weighted imaging
