# Supplementary material for: Saliency-based 3D convolutional neural network for categorising common focal liver lesions on multisequence MRI
Source: Insights Imaging. 2021 Nov 24;12:173. doi: 10.1186/s13244-021-01117-z (PMC8613326; doi:10.1186/s13244-021-01117-z)

**Additional file 1**

**Saliency-based 3D convolutional neural network for categorizing common focal liver lesions at multi-sequence MRI**

**Additional file 1: Fig. S1** Flowchart showing study selection according to the inclusion and exclusion criteria, from initial patient search to training-validation and test dataset randomization


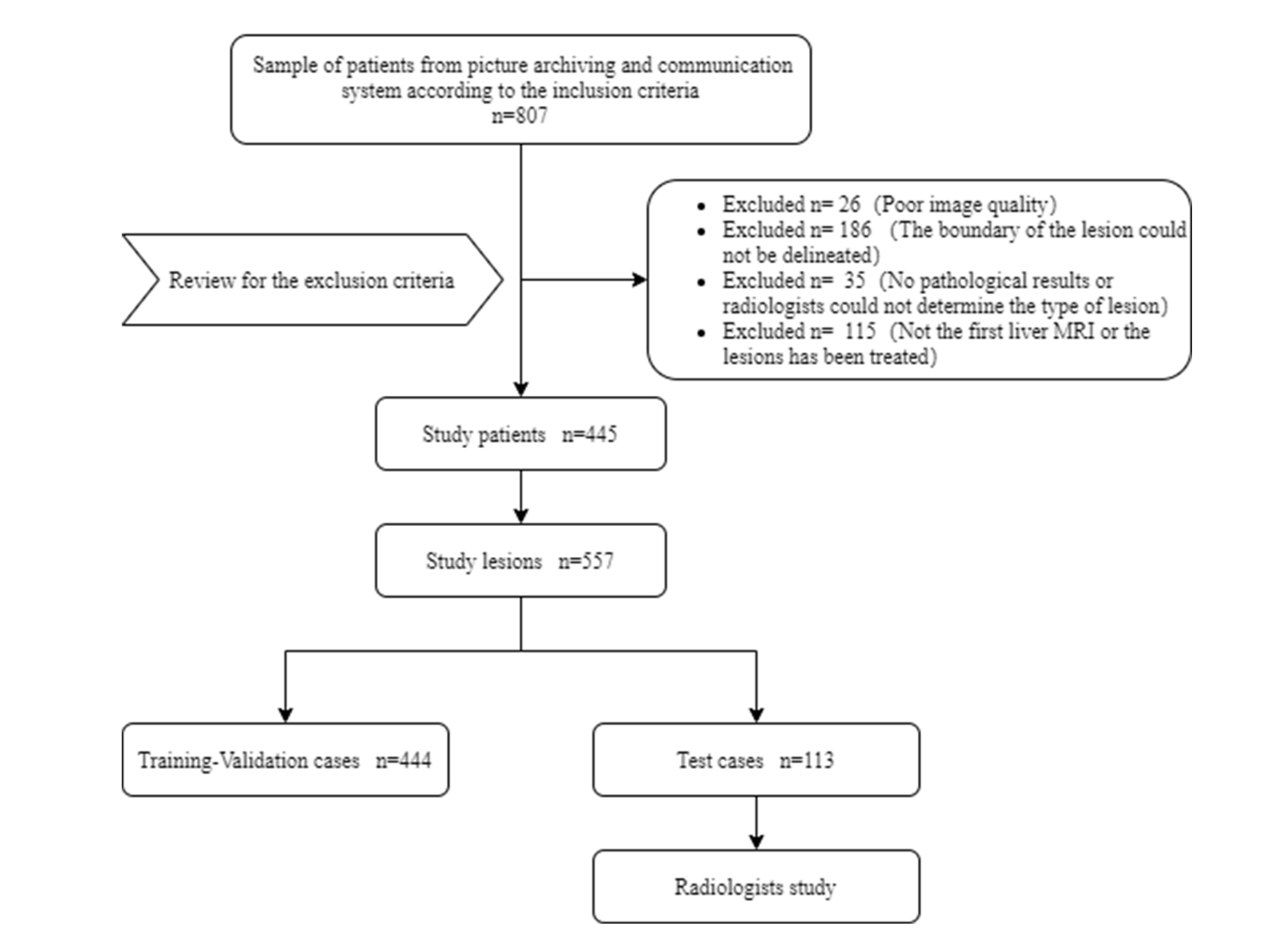


**Additional file 1: Fig. S2** Axial MRI of a 55-year-old man with an abscess. (a) T2WI showed an irregularly shaped, hyperintense neoplasm in segment V. The lesion showed (b) hyperintensity on DWI (c) with a low ADC, (d) hypointensity on T1WI, and (e) targetoid rim enhancement in the LAP, (f) PVP and (g) DP. The lesion was mistaken as ICC by the AI model


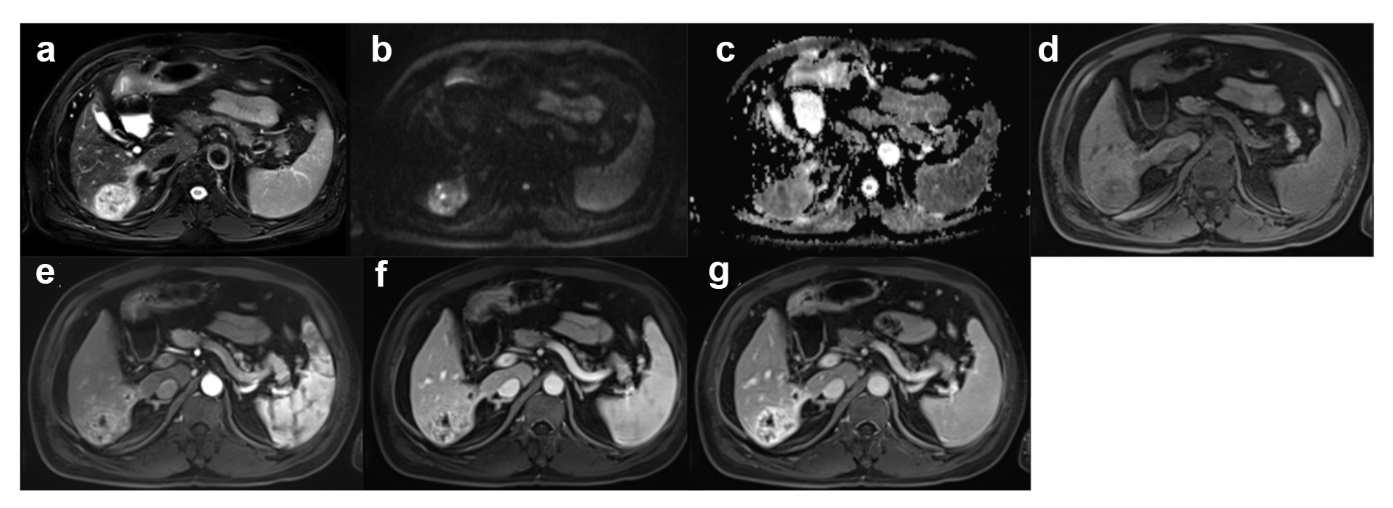

Supplement: Supplementary file 1 — Additional file 1: Figure S1. Flowchart showing study selection according to the inclusion and exclusion criteria, from initial patient search to training-validation and test dataset randomisation. Figure S2 Axial MRI of a 55-year-old man with an abscess. (a) T2WI showed an irregularly shaped, hyperintense neoplasm in segment V. The lesion showed (b) hyperintensity on DWI (c) with a low ADC, (d) hypointensity on T1WI, and (e) targetoid rim enhancement in the LAP, (f) PVP and (g) DP. The lesion was mistaken as ICC by the AI model. [file 13244_2021_1117_MOESM1_ESM.docx]
